# Imaging findings in 75 pediatric patients with pancreaticobiliary maljunction: a retrospective case study

**DOI:** 10.1007/s00383-012-3159-6

**Published:** 2012-08-15

**Authors:** Wan-liang Guo, Shun-gen Huang, Jian Wang, Mao Sheng, Lin Fang

**Affiliations:** 1 Radiology Department, The Children’s Hospital Affliated to Soochow University, Suzhou, 215003 China; 2 Pediatric General Surgery Department, The Children’s Hospital Affliated to Soochow University, Suzhou, 215003 China

**Keywords:** Pancreaticobiliary maljunction, Pediatric, Imaging, Intraoperative cholangiography

## Abstract

**Background:**

Pancreaticobiliary maljunction (PBM) is often associated with congenital choledochal cyst, protein plugs and pancreatitis. Early diagnosis and timely treatment largely depend on imaging. We assessed a series of PBM in children, comparing imaging procedure with histological and pathological findings with regard to diagnosis.

**Methods:**

A retrospective analysis was conducted in 75 pediatric patients with PBM. PBM was defined as common channel at >5 mm. Two radiologists assess the shape of the bile duct and gallbladder, pancreatitis, surgical pathology, symptom profiles, operative notes and pathological records were compared with the imaging findings.

**Results:**

Dilatation of the bile duct was detected in 45 subjects out of the 46 subjects who underwent computed tomography (CT) and nine was diagnosis as PBM. Forty out of 41 subjects were revealed bile duct dilatation in ultrasonography (US). Bile duct dilatation was seen in 59 out of 60 subjects receiving magnetic resonance cholangiopancreatography (MRCP) and 39 were diagnosed as PBM. Seventy-four out of 75 subjects successfully underwent intraoperative cholangiography (IOC); a diagnosis of PBM was established in 60 cases based on IOC alone. The diagnosis rate of pediatric PBM varied significantly among the four groups (*P* < 0.0001). Pair-wise comparison showed a significant difference between the groups of MRCP and CT (*P* < 0.0001), MRCP and US (*P* < 0.0001), IOC and CT (*P* < 0.0001), IOC and US (*P* < 0.0001), CT and US (*P* = 0.0027), and there is no significant difference between the groups of IOC and MRCP (*P* = 0.0502).

**Conclusion:**

US, IOC, CT and MRCP are valuable in showing dilatation of the bile duct and complications in pediatric PBM. MRCP is non-invasive, gives clear views of the pancreaticobiliary junction and should be the first choice for the diagnosis of PBM in children.

## Introduction

Pancreaticobiliary maljunction (PBM) is a rare anomaly, caused by the union of pancreatic duct and bile duct outside the duodenal wall. Under this condition, the sphincter of Oddi can not regulate the outflow of bile and pancreatic juice, leading to two-way regurgitation [1, 2]. Frequent passage of pancreatic juice into the bile duct damages the epithelium of the biliary tract [3]. Bile reflux into the pancreatic duct could lead to pancreatitis.

The clinical presentation of PBM varies considerably, and may include abdominal mass, jaundice, vomiting, pancreatitis, and recurrent abdominal pain. Imaging plays an important role in the diagnosis. The aim of this study was to review a series of clinical and imaging findings in pediatric PBM patients.

## Materials and methods

This study was approved by the Institutional Review Board of our Hospital. Informed consent was signed by the guardians of each child. We retrospectively reviewed the medical records of 75 patients with PBM (24 males; 51 females; age range 2 months to 13 years) who were hospitalized between January 2002 and December 2011. The gold standard for the selection of PBM patients was defined as >5 mm common channel as detected by MRCP, intraoperative cholangiography and CT [4]. PBM was classified based on Komi’s method [5] to the following three types: type I joining of common bile duct with pancreatic duct at approximately 90°, type II joining of pancreatic duct with common bile duct usually at 90°, type III complex arrangement of pancreatic duct and terminal portion of common bile duct.

All patients underwent intraoperative cholangiography. Computed tomography (CT; Philips, Amsterdam, Netherlands) was performed using the anteroposterior projection with the child lying supine. The CT protocols and technical parameters included 5 mm collimation at 10 mm intervals. Ultrasonography (US) (LOGIQ 5 PRO; GE Healthcare, Little Chalfont, UK) was carried out with 5–12 MHz curved and linear abdominal transducers. Magnetic resonance cholangiopancreatography (MRCP) was performed (under sedation for subjects at <10 years of age) on a Symphony 1.5 T scanner (Siemens, Erlangen, Germany) with an abdominal phased array coil under the following modes: T1-weighted and T1-weighted fast spin series (field of view 24–28 cm, repetition time [TR] 173 ms, echo time [TE] 2.64 ms, flip angle 70, matrix 256 × 128, radiofrequency (RF) bandwidth 260 Hz/Px) and a T2-weighted sequence (TR 1,000 ms, TE 60 ms, RF bandwidth 230 Hz/Px). For MRCP, half fourier acquisition single shot turbo spin echo (HASTE) was used with multilayer thin coronal and axial T2-weighted imaging (TR 1,200 ms, TE 80 ms, slice thickness 4 mm). Oblique thick slabs were acquired in the planes of the common bile duct and pancreatic duct. For multi-angle imaging, TR was 4,500 ms, TE 950 ms and slice thickness 60 mm.

Two radiologists who were unaware of the pathological findings independently reviewed the images and reached consensus through discussion. A diagnosis of PBM was established if the common channel is longer than 5 mm. They also assess the shape of the intrahepatic bile duct and gallbladder, pancreatitis, surgical pathology, symptom profiles, operative notes and pathological records were compared with the imaging findings.

### Statistical analysis

Data are presented as number (*n*) and percentage. Univariate comparisons were made using Chi-squared (χ^2^), or Fisher’s exact tests, dependent on statistical distributions, with statistical analysis software (SAS) 8.1. Probability values of *P* < 0.05 were considered statistically significant.

## Results

The morphological type of bile duct dilatation was cystic in 64 patients and fusiform in ten. The bile duct was not dilated in the remaining one subject. Based on Komi’s classification [5], the type of PBM was pancreatic-biliary (type I) in 58 cases, one was type III and biliary-pancreatic (type II) in the remaining 16 cases. Seventy cases underwent surgical resection and five cases underwent secondary cyst excision after external drainage.

Gallbladder and choledochal cyst specimens were stained with hematoxylin and eosin, and examined under a microscope in all 75 subjects. Subserosal fibrosis was seen in all subjects, cirrhosis in four, chronic cholecystitis in 44 and chronic cholangitis in 46.

The most common symptom was abdominal pain (80.0 %), followed by jaundice (20.0 %), abdominal mass (9.3 %), fever (9.3 %), vomiting (9.3 %), and clay-colored stool (6.7 %). One case had biliary rupture and ten had acute pancreatitis. Amylase level in the bile was determined in 51 subjects, and ranged from 2,558 to 148,854 U/L, with an average of 47,373 U/L. Clinicopathological characteristics and the correlation of different types with clinical manifestation are summarized (Tables 1, 2).

**Table 1:** Clinicopathological characteristics of 75 pediatric patients with PBM

Variable	Total	Infant group (≤1 year, *n* = 22)	Classical pediatric group (>1 year, *n* = 53)	*P* value
Age	(2 months to 13 years)	(2 months to 1 year)	(1.2 to 13 years)	–
Sex (male/female)	75	6/16	19/35	0.5055
PBM type				
Type I	58	16	42	0.5393
Type II	16	10	6	<0.001
Type III	1	–	1	1.0000
Clinical manifestation				
Abdominal pain	60	14	46	0.0303
Jaundice	15	10	5	0.0012
Fever	7	2	5	1.0000
Vomiting	7	3	4	0.4116
Abdominal mass	7	3	4	0.4116
Clay-colored stool	5	3	2	0.1469
Complication				
Chronic cholecystitis	44	9	35	0.0442
Acute pancreatitis	13	5	8	0.4266
Protein plugs	10	3	7	0.9604
Biliary rupture	1	1	–	0.2933

**Table 2:** The correlation of different types with clinical manifestation

Symptoms	(Type I, *n* = 58)	(Type II, III, *n* = 17)	*P* value
Abdominal pain	47 (81.0 %)	13 (76.5 %)	0.7340
Jaundice	11 (18.9 %)	4 (23.5 %)	0.7340
Abdominal mass	5 (8.6 %)	2 (11.7 %)	0.6533
Vomiting	6 (10.3 %)	1 (5.8 %)	1.0000
Fever	5 (8.6 %)	2 (11.7 %)	0.6533
Clay-colored stool	4 (6.8 %)	1 (5.8 %)	0.6131
Acute pancreatitis	13 (22.4 %)	0 (–)	0.0321
Protein plugs	7 (12.0 %)	3 (17.6 %)	0.6855

Fisher’s exact test is used

### Imaging studies

**Fig. 1:**
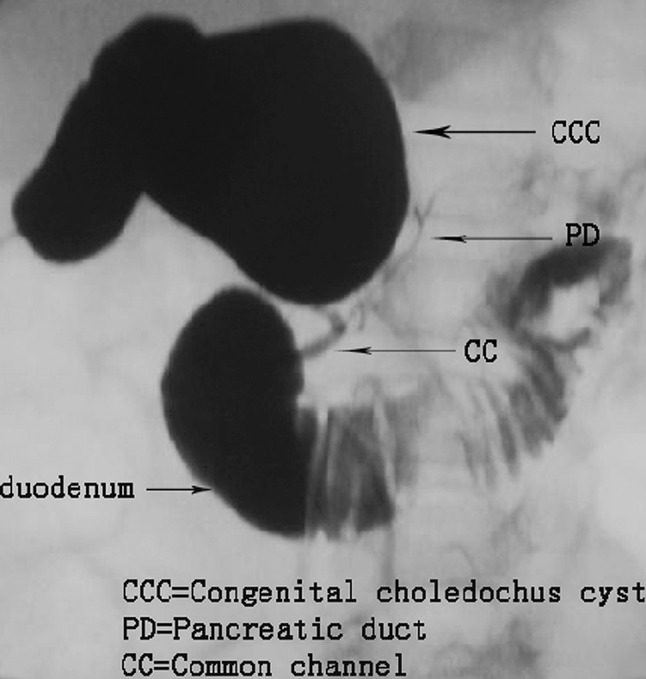
Intraoperative cholangiography shows the junction of the bile and pancreatic ducts located outside the duodenal wall. The common bile duct joins pancreatic duct (type I)

Seventy-four of the 75 subjects underwent successful intraoperative cholangiography. A diagnosis of PBM (Fig. 1) could be established based on cholangiography results alone in 60 subjects. Protein plugs in common bile duct were seen in ten cases. In one subject, bile leakage was found during the procedure.

**Fig. 2:**
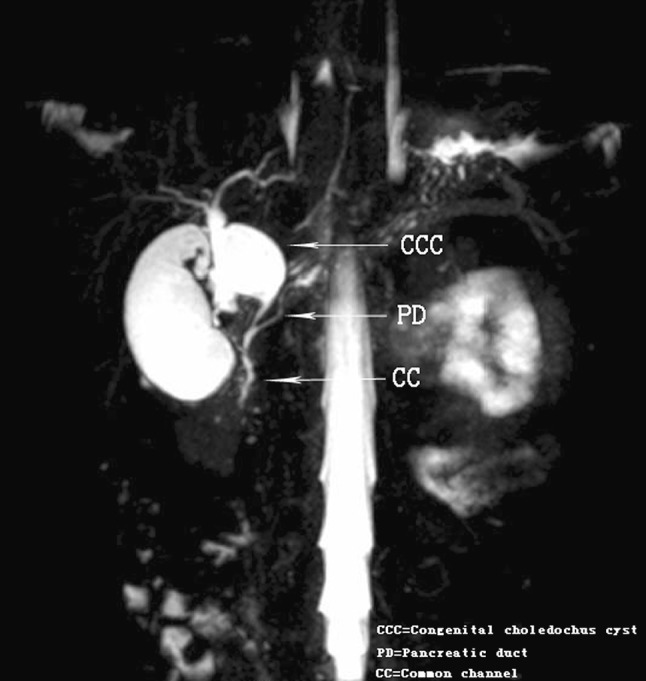
Magnetic resonance cholangiopancreatography. Coronal 4 mm-thick half fourier acquisition single shot turbo spin echo image shows the pancreatic duct joining the common bile duct outside the duodenal wall (type II)

Sixty subjects underwent MRCP. Dilatation of the bile duct was seen in 59 subjects. Dilatation of both common bile duct and intrahepatic bile duct were observed in ten subjects. The main pancreatic duct and PBM (Fig. 2) were seen in 39 subjects. Protein plugs in common bile duct were seen in seven cases. One case of non-dilated PBM was not revealed by either CT or US, but clearly visible in MRCP.

**Fig. 3:**
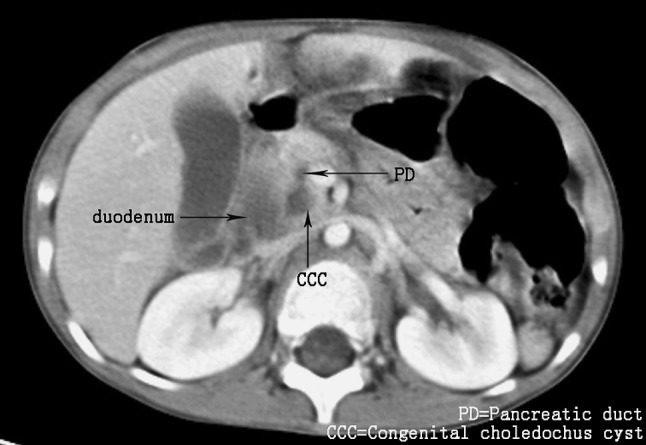
Abdominal enhanced computed tomography, showing convergence of the dilatation of the common bile duct and pancreatic duct

Forty-six subjects underwent CT; dilatation of the bile duct was seen in 45 subjects. Dilatation of the intrahepatic and common bile duct was seen in nine cases. Six patients had cholecystitis; three had protein plugs in the gallbladder, three had protein plugs in the common bile duct. Convergence of the pancreatic and common bile ducts was seen in CT (Fig. 3) in nine cases.

**Fig. 4:**
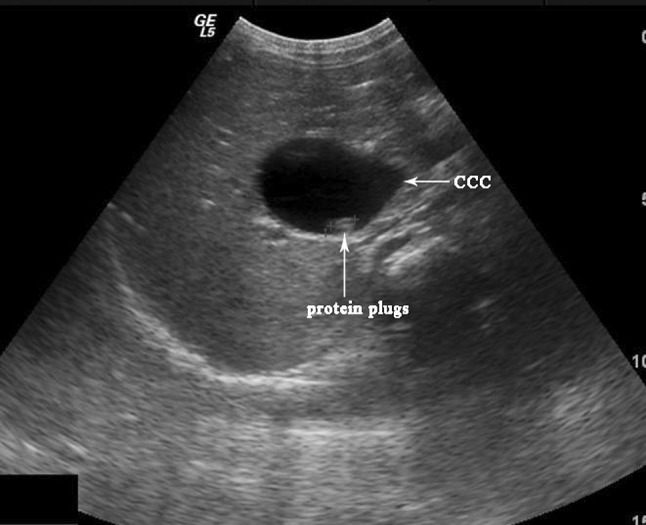
Transverse ultrasonography shows the common duct dilated with protein plugs

Forty-one subjects underwent US; dilatation of the common bile duct was seen in 40. The gallbladder wall was thickened in 24 cases; protein plugs in the dilatation common bile duct was identified in three case (Fig. 4).

The diagnosis rate of pediatric PBM varied significantly among the four groups (*P* < 0.0001). Pair-wise comparison showed a significant difference between the groups of MRCP and CT (*P* < 0.0001), MRCP and US (*P* < 0.0001), IOC and CT (*P* < 0.0001), IOC and US (*P* < 0.0001), CT and US (*P* = 0.0027), and there is no significant difference between the groups of IOC and MRCP (*P* = 0.0502) (Table 3).

**Table 3:** Diagnosis rate of pediatric PBM

	IOC (*n* = 75)	MRCP (*n* = 60)	CT (*n* = 46)	US (*n* = 41)
Diagnosis rate of PBM	60/75 (80.0 %)	39/60 (65.0 %)	9/46 (19.6 %)	–

Chi-squared revealed an overall difference in diagnosis rate of PBM among the four groups (*P* < 0.0001). Pair-wise comparison showed a significant difference between the groups of MRCP and CT (*P* < 0.0001), MRCP and US (*P* < 0.0001), IOC and CT (*P* < 0.0001), IOC and US (*P* < 0.0001), CT and US (*P* = 0.0027), and there is no significant difference between the groups of IOC and MRCP (*P* = 0.0502)

## Discussion

Establishing a diagnosis of PBM is very challenging prior to dilatation of the bile duct. Also, dilatation of the bile duct must be differentiated from choledochal cysts (CCC), in which either extrahepatic bile duct or extrahepatic with intrahepatic bile ducts could be dilated [6]. Imaging results could help in differential diagnosis by identifying the union of the pancreatic duct and bile duct.

Pancreatic sphincter deficiency in PBM could lead to reflux of pancreatic juice into bile duct [7]. High level of amylase in the bile is suggestive of PBM in children. In this series of pediatric PBM patients, biliary amylase was elevated in all cases (51 out of 75) receiving such examination. Recurrent reflux of pancreatic juice into the bile duct causes chronic inflammation of the bile duct and may contribute to the formation of protein plugs. In this study, all subjects had hyperplasia, 44 had chronic cholecystitis and 46 had chronic cholangitis. These findings are consistent with an earlier report by Noda [8]. No cancer in the gallbladder or bile duct was identified despite of increased risk of carcinogenesis, possibly due to young age.

The most common symptom in our series was abdominal pain (80.0 %), followed by jaundice (20.0 %), abdominal mass (9.3 %), fever (9.3 %), vomiting (9.3 %), and clay-colored stool (6.7 %). Some of the clinicopathological characteristics is different between infant group and classical pediatric group (Table 1). Pediatric patients with such symptoms should be suspected of having PBM and should be examined with imaging or endoscopic retrograde cholangiopancreatography (ERCP).

PBM is classified into three types (I, II and III) by Komi [5]. Forty-one cases of choledochal cyst complicated by PBM were reported by Onogawa [9]—58 % type I, 25 % type II and 17 % type III. In our series, 58 cases were type I; 16 cases were type II. One is type III PBM. The small size in type III may be caused by the young age in this series. Type I is more easily to cause acute pancreatitis than the other types (Table 2). Similar to a reported by Kimura [10], all but one cases in our series had CCC on the background of PBM, suggesting a close and interacting etiology of the two conditions.

ERCP is the golden standard for the diagnosis of PBM [11], but requires anesthesia. For pediatric patients, the procedure is much more difficult. As a result, the rate of complications secondary to ERCP is as high as 13.5 % in children [12].

US has clear advantages over other imaging modalities: it is non-invasive and free from irradiation [13]. US could be used to measure the diameter of common bile duct cysts, and can detect cholecystitis and protein plugs. Sugai et al. [14] reported that PBM is often indicated by gallbladder wall thickening measured by US in pediatric patients. Chapuy et al. [13] reported MRCP and US studies in four pediatric patients with PBM; they concluded that although common pancreaticobiliary channel can be identified by US, the method is limited because it does not provide accurate measurement of the common channel since the coronal plane is not visualized. Also, reflux could not be detected by US.

CT has higher resolution than MRCP. Contrast-enhanced CT can differentiate hepatic cyst and intrahepatic/extrahepatic bile duct and pancreatic duct dilatation. In addition to establishing a diagnosis of PBM [15], CT is also sensitive modality to detect potential carcinoma. Convergence of the pancreatic and common bile ducts was clearly manifested and PBM was correctly diagnosed under contrast-enhanced helical computed tomography in nine cases in this series. Conventional CT, however, can not demonstrate the common pancreaticobiliary channel clearly [9]. Watanabe et al. [16] reported the use of infusion cholangiography spiral CT (helical CT during drip infusion cholangiography, DIC-CT) in the diagnosis of PBM. Using an intravenous hepatobiliary agent (iodipamide meglumine) and 3-D CT, high-resolution images were obtained. The pancreaticobiliary channel and two-way regurgitation of pancreatic juice and bile were clearly visible. This technique has not been widely used in children due to exposure to ionizing irradiation.

MRCP is a non-invasive, low risk technique widely used in the diagnosis of pancreatic and biliary anomalies [17, 18]. It provides high-resolution 3-D images of the bile tree and pancreatic duct at multiple positions and from multiple angles. In our series, MRCP could reveal the extrahepatic bile duct, gallbladder, protein plugs and bile duct stenosis, and as such, is helpful in differentiating PBM from CCC. MRCP gives clear views of the pancreaticobiliary junction than CT and US. Its value in diagnosing pediatric PBM is as high as IOC (Table 3). The length of the pancreaticobiliary channel could also be reliably measured by MRCP. Children with PBM maybe have some non-dilated ducts that cannot be found by US or CT. Compared with US and CT, MRCP could provide improved visualization of non-dilated ducts in PBM accompanied by common channel protein plugs [19], although the body and tail of the pancreatic duct are often not clear. Chu et al. [20] found that lemon/orange juice can improve the view of the pancreatic duct. Reflux of pancreatic juice into the bile duct could be observed by dynamic MRCP when pancreatic exocrine secretion is stimulated with secretin [21, 22]. Increasing evidence suggest that MRCP is almost equivalent to ERCP in terms of the information it could provide [23].

Intraoperative cholangiography is used during surgery to observe the anatomy of the pancreaticobiliary system and the function of Oddi sphincter. It could also be used to assess distal bile duct patency [24]. The technical success rate of cholangiography was 98.7 % in our series. PBM was seen in 60 out of 75 cases. However, the common channel was not clearly visible in some patients who may have had a large choledochal cyst or a forwardly placed duodenum.

In summary, all four imaging modalities (US, IOC, CT and MRCP) are valuable in showing dilatation of the bile duct and complications in pediatric PBM. MRCP is non-invasive, gives clear views of the pancreaticobiliary junction and should be the first choice for the diagnosis of PBM in children.

## References

[1] Kamisawa T, Egawa N, Nakajima H, Tsuruta K, Okamoto A, Matsukawa M (2005). Origin of the long common channel based on pancreatographic findings in pancreaticobiliary maljunction. Dig Liver Dis.

[2] Kamisawa T, Tu Y, Nakajima H, Egawa N, Tsuruta K, Okamoto A (2007). The presence of a common channel and associated pancreaticobiliary disease: a prospective ERCP study. Dig Liver Dis.

[3] Tanno S, Obara T, Fujii T, Mizukami Y, Shudo R, Nishino N, Ura H, Klein-Szanto AJ, Kohgo Y (1998). Proliferative potential and K-ras mutation in epithelial hyperplasia of the gallbladder in patients with anomalous pancreaticobiliaryductal union. Cancer.

[4] Guelrud M, Morera C, Rodriguez M, Prados JG, Jaén D (1999). Normal and anomalous pancreaticobiliary union in children and adolescents. Gastrointest Endosc.

[5] Komi N (1991). New classification of anomalous arrangement of the pancreaticobiliary ducts (APBD) in choledochal cyst: a proposal of new Komi’s classification of APBD (in Japanese). J Jpn Pancr Soc.

[6] Hung MH, Lin LH, Chen DF, Huang CS (2011). Choledochal cysts in infants and children: experiences over a 20-year period at a single institution. Eur J Pediatr.

[7] Anderson MC, Hagstrom WJ (1962). A comparison of pancreatic and biliary pressures recorded simultaneously in man. Can J Surg.

[8] Noda Y, Fujita N, Kobayashi G, Ito K, Horaguchi J, Takasawa O, Obana T, Ishida K, Senoo S, Yonechi M, Suzuki T, Hirasawa D, Sugawara T, Kobari M, Sawai T, Uzuki M, Watanabe M (2007). Histological study of gallbladder and bile duct epithelia in patients with anomalous arrangement of the pancreaticobiliary ductal system: comparison between those with and without a dilated common bile duct. J Gastroenterol.

[9] Onogawa T, Rikiyama T, Unno M, Matsuno S (2007) Biliopancreatic maljunction: classification, diagnosis, and treatment. In: diseases of the pancreas. Springer-Verlag Berlin and Heidelberg GmbH and Co. KG, Berlin, pp 895–901

[10] Kimura W (2009). Congenital dilatation of the common bile duct and pancreaticobiliary maljunction—clinical implications. Langenbecks Arch Surg.

[11] Bheerappa N, Sastry RA (2001). Pancreaticobiliary ductal union (review). Trop Gastroenterol.

[12] Paris C, Bejjani J, Beaunoyer M, Ouimet A (2010). Endoscopic retrograde cholangiopancreatography is useful and safe in children. J Pediatr Surg.

[13] Chapuy S, Gorincour G, Roquelaure B, Aschero A, Paris M, Lambot K, Delarue A, Bourlière-Najean B, Petit P (2006). Sonographic diagnosis of a common pancreaticobiliary channel in children. Pediatr Radiol.

[14] Sugai M, Ishido K, Endoh M, Hada R, Munakata H (2010). Sonographic demonstration of wall thickness of the gallbladder in pediatric patients with pancreatico-biliary maljunction. J Hepatobiliary Pancreat Sci.

[15] Ono S, Fumino S, Iwai N (2011). Diagnosis and treatment of pancreaticobiliary maljunction in children. Surg Today.

[16] Watanabe Y, Kubota H, Honma T, Hosoya T, Yamaguchi K (1997) Usefulness of helicalD IC-CT in pancreaticobiliary maljunction. Nippon Igaku Hoshasen Gakkai Zasshi (Japanese) 57: 249–52 9164113

[17] Anupindi SA (2008). Pancreatic and biliary anomalies: imaging in 2008. Pediatr Radiol.

[18] Kamisawa T, Tu Y, Egawa N, Tsuruta K, Okamoto A, Kamata N (2007). MRCP of congenital pancreaticobiliary malformation. Abdom Imaging.

[19] Schindera ST, Merkle EM (2007) MR cholangiopancreatography: 1.5T versus 3T. Magn Reson Imaging Clin N Am 15(3):355–364, vi-vii 10.1016/j.mric.2007.06.009 17893055

[20] Chu ZQ, Ji Q, Zhang JL (2010). Orally administered lemon/orange juice improved MRCP imaging of pancreatic ducts. Abdom Imaging.

[21] Sai JK, Suyama M, Kubokawa Y, Tadokoro H, Sato N, Ookubo H, Iida Y, Kojima K (2003). Occult pancreatobiliary reflux in patients with a normal pancreaticobiliary junction. Gastrointest Endosc.

[22] Motosugi U, Ichikawa T, Araki T, Kitahara F, Sato T, Itakura J, Fujii H (2007). Secretin-stimulating MRCP in patients with pancreatobiliary maljunction and occult pancreatobiliary reflux: direct demonstration of pancreatobiliary reflux. Eur Radiol.

[23] Sugiyama M, Baba M, Atomi Y, Hanaoka H, Mizutani Y, Hachiya J (1998). Diagnosis of pancreatobiliary junction: value of magnetic resonance cholangiopancreatography. Surgery.

[24] Fujisaki S, Tomita R, Koshinaga T, Park E, Kimizuka K, Shibata M, Fukuzawa M, Nemoto N (2003). The clinicopathological studies on patients with pancreaticobiliary maljunction that was detected by intraoperative cholangiography under laparoscopic cholecystectomy. Hepatogastroenterology.

